# A focus on 1-azahomocubane: the new kid on the block

**DOI:** 10.1039/d3sc90114a

**Published:** 2023-07-07

**Authors:** Cecile Elgindy, Mark D. Levin

**Affiliations:** a Department of Chemistry, University of Chicago Chicago IL 60637 USA marklevin@uchicago.edu

## Abstract

Strained hydrocarbons have recently regained interest as potential drug candidates. However, the study of their heteroatom analogs has remained limited, despite differing by only a single atom. The first synthesis of 1-azahomocubane by Williams, Eaton and co-workers (T. Fahrenhorst-Jones *et al.*, *Chem. Sci.*, 2023, **14**, 2821–2825, https://doi.org/10.1039/D3SC00001J) is discussed within the context of nitrogen scanning of strained hydrocarbons.

## Abstract 2

A highlight of the first synthesis of 1-azahomocubane and initial studies of this molecule (https://doi.org/10.1039/D3SC00001J), presenting opportunities for nitrogen scanning of strained hydrocarbon molecules.

## Graphical abstract

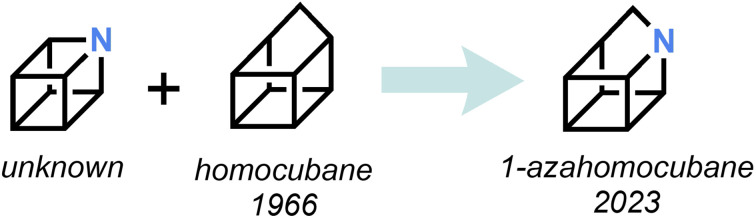

The exchange of a single atom can dramatically alter the properties of a molecule. Reactivity, biological activity, stability, selectivity, physical properties, and synthetic tractability of the molecule can all be impacted, simply by the substitution of one atom for another. Such effects have been observed in natural products and drug candidates, exemplified by the “necessary nitrogen effect” in medicinal chemistry whereby the replacement of a carbon atom for a nitrogen atom in an aromatic system can profoundly alter the molecule’s pharmacological profile.^1,2^

Meanwhile, renewed interest in strained hydrocarbons has arisen from their potential as benzene bioisosteres, which increase the parent molecule’s three-dimensionality without introducing substantial metabolic hotspots, owing to the high s character of the exocyclic C–H bonds.^3^ Cubane, bicyclo[1.1.1]pentane, and bicyclo[1.1.2]hexane have all been proposed as attractive substructures for drug candidates along these lines, which has led to a flurry of activity examining the functionalization of these scaffolds, with a particular focus on C–H functionalisation and strain release methods for their synthesis.^4–6^

One can imagine, in analogy to the necessary nitrogen effect for benzenoid systems, that the corresponding heterocyclic series would be valuable in the strained hydrocarbon family of compounds. Surprisingly, however, very few of the aza-analogs of these hydrocarbons are known, with two azahomocubanes and a small number of substituted azabicyclohexanes previously reported, mostly of the 2-azabicyclohexane class (Fig. 1).^7–9^

**Fig. 1:**
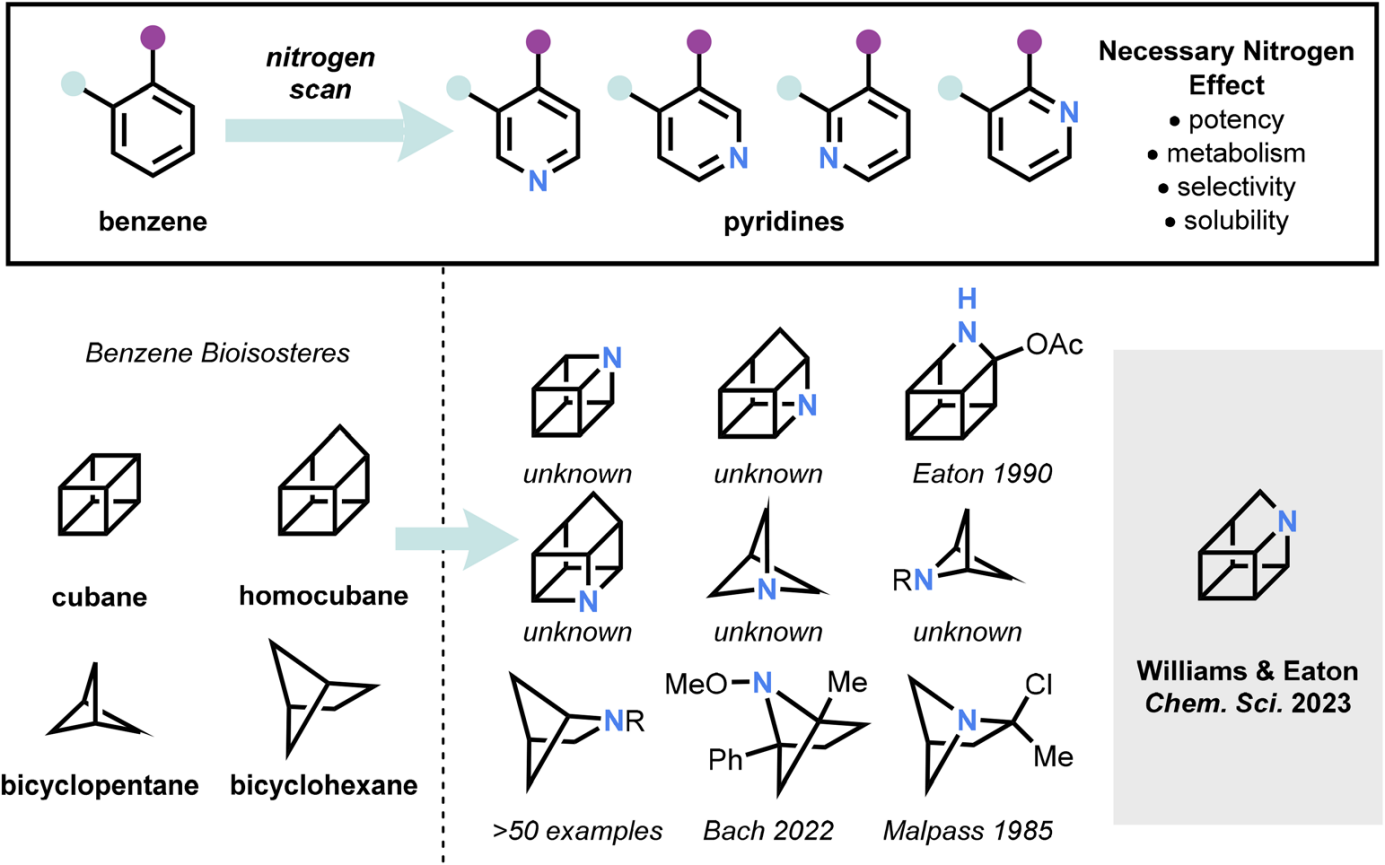
The necessary nitrogen effect in benzenoid systems, imagined for benzene bioisosteres.

Williams, Eaton, and co-workers disclose the first synthesis of 1-azahomocubane, an aza-analog of homocubane (https://doi.org/10.1039/D3SC00001J).^10^ The synthesis and study of this surprisingly stable, strained molecule is a rare foray into the azahomocubane series, providing a useful comparison to related structures and enabling insights into this underexplored structural space.

The synthesis pinpointed azidyl cubane as a strategic intermediate that enabled the incorporation of nitrogen into the core of the strained ring framework, building upon previous reports of a substituted 9-azahomocubane.^11^ A sequence of rearrangements beginning from the azidyl cubane was initiated by strong acid followed by *N*-chlorination, resulting in nitrogen incorporation into the ring system. To complete the synthesis, functional group manipulation enabled the requisite ring closure *via N*-alkylation to generate the parent 1-azahomocubane, which was trapped *in situ* as a salt to simplify its isolation (Fig. 2).

**Fig. 2:**
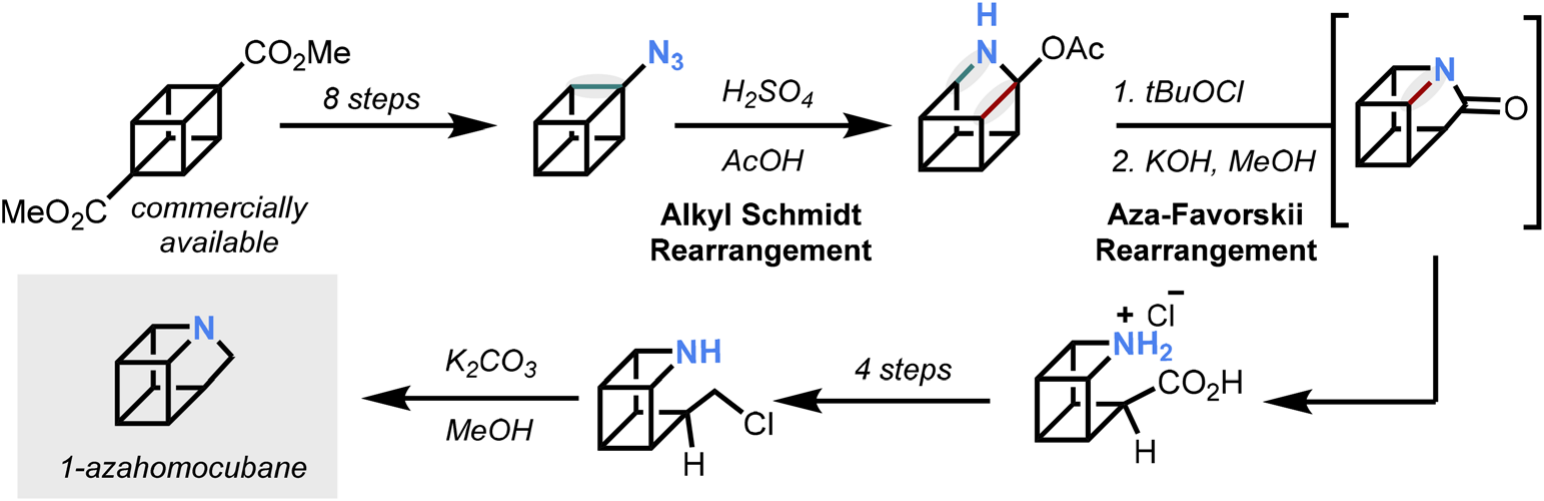
Synthesis of 1-azahomocubane.

With the target compound in hand, a preliminary survey of chemical and physical properties could be conducted. The free amine was found to be stable as a solution over months, whereas the hydrochloride salt was observed to undergo chloride mediated ring-opening. 1-Azahomocubane was calculated to be more stable than homocubane and was observed to be less reactive towards Ag^+^ mediated isomerisations, suggesting that nitrogen-atom exchange enhances the stability of the strained ring system. Indeed, an X-ray structure of the bis(azahomocubane) silver(i) perchlorate salt was collected. Finally, azahomocubane was found to be meaningfully less basic than related polycyclic tertiary amines, with the additional strain likely inducing increased lone pair s-character.

This work by Williams, Eaton and co-workers highlights an underexplored region of chemical space. The synthesis of strained polycyclic heterocycles conceptually derived from benzene bioisosteres is not without challenges, but this work will hopefully spur further development in this area.

## Author contributions

All authors contributed equally.

## Conflicts of interest

There are no conflicts to declare.

## Supplementary Material
